# IL-33 citrine reporter mice reveal the temporal and spatial expression of IL-33 during allergic lung inflammation

**DOI:** 10.1002/eji.201242863

**Published:** 2012-11-21

**Authors:** Clare S Hardman, Veera Panova, Andrew N J McKenzie

**Affiliations:** MRC Laboratory of Molecular BiologyHills Road, Cambridge, UK

**Keywords:** Asthma, ILC2, Interleukin-33 (IL-33), Nuocytes, Type-2 pneumocytes

## Abstract

Interleukin-33 (IL-33) is an IL-1 family cytokine that signals via its receptor T1/ST2, and is a key regulator of inflammation, notably the type-2 response implicated in allergic asthma. Critical to our understanding of the role of IL-33 is the identification of the cellular sources of IL-33. Although progress has been made in this area, the development of a robust live cell reporter of expression would allow the localisation of IL-33 during ongoing immune responses. We have generated a fluorescent reporter mouse line, *Il33^Cit/+^*, to define the expression profile of IL-33 in vivo and demonstrate its temporal and spatial expression during experimental allergic asthma responses. We found that type-2 pneumocytes constitute the major source of IL-33 upon allergic lung inflammation following exposure to OVA, fungal extract or ragweed pollen. Using *Il33^Cit/Cit^* mice (IL-33-deficient), we establish a role for IL-33 early in the initiation of type-2 responses and the induction of nuocytes (ILC2). We also demonstrate a potential mechanism of action by which IL-33 rapidly initiates type-2 immune responses. *Il33^Cit/+^* mice have enabled new insights into the initiation of type-2 responses and will provide an important tool for further dissection of this important inflammatory pathway in vivo.

## Introduction

Type-2 immune responses are characterised by the production of the cytokines IL-4, IL-5 1, IL-9 2 and IL-13 3 and constitute the host's defence against helminthic worm infections. Type-2 cytokines act to induce inflammation, cellular infiltration, smooth muscle contraction, goblet cell hyperplasia, mucus production and increased serum IgE 4 in order to expel the parasite 5. Notably, allergic asthma results from inappropriate activation of the type-2 immune responses in the lung triggered by contact with innocuous allergens, such as pollen. In the allergic lung, the type-2 response leads to the respiratory distress characteristic of asthma 6.

Recently, IL-33 and IL-25 have been shown to act upstream of the type-2 effector cytokines as crucial initiators of type-2 responses. Consistent with this, allergic asthma sufferers express higher levels of IL-33 and IL-25 than healthy subjects 7–10. IL-33 is a member of the IL-1 family of cytokines but, unlike IL-1α, IL-1β, and IL-18 that require caspase-1 processing for biological activity, IL-33 is inactivated by caspase-1 cleavage 11–13. IL-33 binds to the receptor T1/ST2 via its C terminal region and thereby initiates a type-2 immune response 10. In activating this response, IL-33 shares a level of redundancy with IL-25, although the exact nature of this functional overlap is not well understood. T1/ST2 is present on subsets of Th2 cells which, when activated, produce type-2 cytokines 1. However, Th2 cells are not alone in the propagation of the type-2 response, and more recently the role of a number of innate cells has been recognised. Nuocytes 14–18, basophils 19, mast cells and eosinophils express the receptors for and respond to IL-33 and IL-25, either in the production of type-2 cytokines or through their recruitment and activation 5,19. The means by which IL-33 is secreted to induce these responses is unknown since IL-33 does not contain a signal peptide for secretion via the classical ER-Golgi body pathway. The prevailing hypothesis is that IL-33 is released in a passive manner as a result of necrosis or cell damage, and as such acts as an alarmin to elicit a protective immune response. During necrosis IL-33 remains in its active form 13, however, under apoptotic conditions caspases 3 and 7 cleave IL-33 rendering it inactive, presumably as a protective measure to prevent unwanted inflammation in response to programmed cell death 11. Interestingly, IL-33 acts not only as a cytokine, but also as a chromatin-binding factor. The targets and exact function of IL-33 in the nucleus remain to be elucidated, though it is thought that IL-33 may exhibit transcriptional repressor activity 20.

It is currently thought that, upon contact with airborne allergens and pathogens, the airways epithelium initiates a type-2 response through the release of IL-33 and IL-25 21. Structural epithelial cells, endothelial cells and smooth muscle cells are reported to produce IL-33 in the lung 7,22, potentially in response to TLR signalling 23. Similarly, Wills-Karp et al. 24 describe expression of IL-33 by macrophages, as well as lung epithelial cells and DCs, in mice infected with *Nippostrongylus brasiliensis*. Using an IL-33 LacZ reporter mouse strain, Pichery et al. 25 recently demonstrated that IL-33 is constitutively expressed in a variety of epithelial barrier tissues, including those of the vagina, lung and salivary glands. This report also showed that, unlike in human tissues, murine endothelial cells do not express IL-33.

IL-33 has been linked to a number of inflammatory disorders including allergic asthma, rheumatoid arthritis, allergic rhinitis and ulcerative colitis. It is important to elucidate when and where IL-33 is produced in these conditions, since this information could provide new insights into disease aetiology and the development of novel treatments for these inflammatory disorders. Although valuable, the nature of LacZ reporter systems means that reporter activity can only be visualised on cryosections. To analyse IL-33 expression directly in vivo we therefore generated *Il33Citrine* (*Il33^Cit/+^)* reporter mice. We established that type-2 pneumocytes represent the preeminent source of IL-33 in the lungs of naïve mice and those challenged with inducers of allergic lung inflammation. Furthermore, kinetic analyses of IL-33 production revealed that this cytokine plays a role early in the induction of type-2 responses and might be responsible for the amplification of the response through paracrine upregulation of its own expression.

## Results

### Constitutive expression of IL-33 promoter-driven citrine

The mode of action of IL-33 as a cytokine or nuclear factor is not clear. To identify cellular sources of IL-33 an *Il33^Cit^*^/+^ reporter mouse line was generated (Supporting Information Fig. 1A). The reporter uses citrine fluorescence as a surrogate for IL-33 mRNA expression with the GFP-derived *Citrine* gene inserted, using gene targeting, directly downstream of the ATG start codon of *Il33* 25. Analysis of these mice by flow cytometry showed that citrine is present constitutively in lung tissue (Supporting Information Fig. 1B). The majority of which were CD45^−^ non-haematopoietic cells (Supporting Information Fig. 1B). Of these citrine^+^CD45^−^ cells 40–50% were positive for the epithelial cell marker EpCam (Supporting Information Fig. 1B). Analysis of the naïve mediastinal lymph node revealed (Supporting Information Fig. 1C), that these citrine^+^ cells were non-haematopoetic cells and probably represent fibroblastic reticular cells (staining positively for VCAM-1, data not shown) consistent with a previous report 26.

### Induction of IL-33 promoter-driven citrine expression in experimental allergic lung inflammation

**Figure 1:**
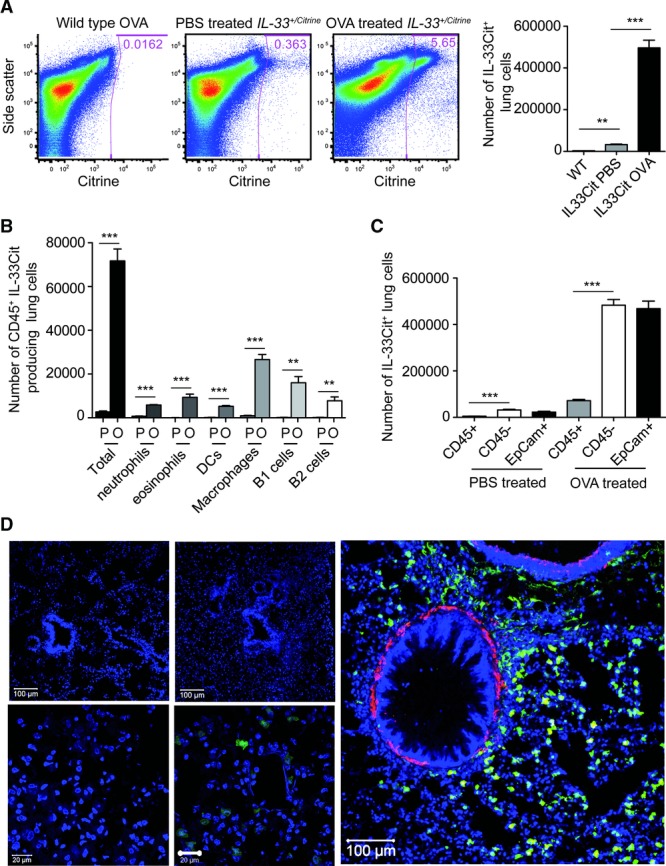
OVA significantly upregulates IL-33 promoter expression. (A) Flow cytometry plots of lung samples from *Il33^Cit/+^* mice treated with either PBS or OVA are shown. (B) Expression of citrine by haematopoietic cells was determined by flow cytometry (P: PBS, O: OVA). (C) Expression of haematopoietic and epithelial cell markers by citrine^+^ lung cells from OVA and PBS-treated mice were determined by flow cytometry. (A–C) Data are shown as mean + SEM of 3–6 mice and are representative of three independent experiments. **p < 0.01; ***p < 0.001, unpaired Student's *t*-test. (D) A confocal image of a cryosection of lung tissue from a WT mouse treated with PBS, stained with DAPI is shown (blue, top left, 20× original magnification). A confocal image of a cryosection of lung tissue from a WT mouse treated with OVA, stained with DAPI, is shown with increased gain (bottom left, 63× original magnification). A confocal image of a cryosection of lung tissue, from an *Il33^Cit/+^* (green) mouse treated with PBS, stained with DAPI is shown (top middle, 20× original magnification). A confocal image of a cryosection of lung tissue, from an *Il33^Cit/+^* mouse treated with PBS, stained with DAPI is shown with increased gain (bottom middle, 63× original magnification). A confocal image of a cryosection of lung tissue from an *Il33^Cit/+^* (green) mouse treated with OVA, stained with DAPI and smooth muscle actin (red) is shown (far right, 63× original magnification). Data shown are from one experiment with five mice representative of three independent experiments; images are consistent for a minimum of three micrographs per mouse.

To investigate the sources of IL-33 in vivo during experimental allergic lung inflammation, the OVA model 14 was used. A profound induction of citrine was observed upon OVA challenge; analysis by flow cytometry measured, on average, a 15-fold increase in citrine levels over those observed in the PBS-treated group (Fig. 1A). Very few CD45^+^ citrine^+^ cells were observed in the PBS-treated mice (approximately 2 500 cells) made up of macrophages (Gr-1^−^/SiglecF^−^/CD11b^+^/CD11c^+/−^) and neutrophils (Gr-1^hi^/SiglecF^−^/CD11b^−^/CD11c^−^) and a very small fraction of DCs (Gr-1^−^/SiglecF^−^/CD11b^−^/CD11c^+^). In contrast mice treated with OVA had a significant population of CD45^+^ citrine^+^ cells (approximately 70 000 cells), an increase of approximately 28-fold upon OVA treatment. Of the cell types investigated macrophages, eosinophils (Gr-1^−^/SiglecF^+^/CD11b^−^/CD11c^−^) and B cells account for most CD45^+^ citrine expression (Fig. 1B). However, 80–90% of the citrine^+^ cells were CD45^−^ and the majority of those were CD45^−^EpCam^+^ epithelial cells (Fig. 1C). Strikingly, upon OVA-induced allergic lung inflammation 15–20% of all EpCam^+^ lung cells expressed citrine, in comparison with 1% in PBS-treated mice.

**Figure 2:**
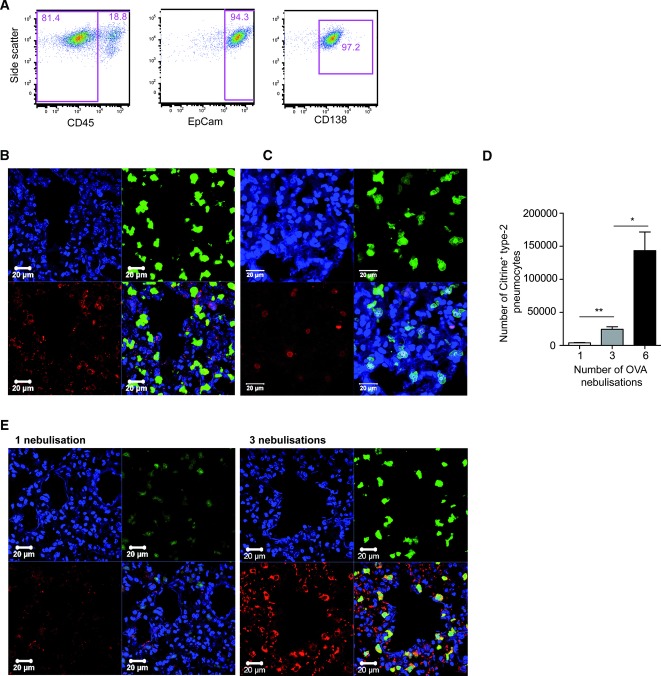
Kinetic study of IL-33 promoter expression. (A) IL-33 expression was analysed by gating on citrine^+^ cells and assessing type-2 pneumocyte surface marker (CD45^−^, EpCam^+^ CD138^+^) expression on CD45^−^ citrine^+^ cells. Data are representative of four independent experiments each performed with *n* = 5 mice. (B) A confocal image of a cryosection of lung tissue from an *Il33^Cit/+^* (green) mouse treated with OVA, stained with DAPI (blue) and SPC (red) to mark type-2 pneumocytes is shown. (C) A confocal image of a cryosection of lung tissue from an *Il33^Cit/+^* (green) mouse treated with OVA, stained with DAPI (blue) and anti-IL-33 antibody (red) is shown. (B, C) Original magnification 63×. Scale bar = 20 μm. (D) The expression of citrine by type-2 pneumocytes after treatment with OVA was determined by flow cytometry, and shown as mean +SEM of *n* = 5 mice. Data shown are representative of three independent experiments. *p < 0.1; **p < 0.01, unpaired Student's *t*-test. (E) Confocal images of cryosections of lung tissue, from an *Il33^Cit/+^* (green) mouse treated with either one or three OVA nebulisations after sensitisation, stained with DAPI (blue) and SPC (red) are shown at 63× original magnification. Scale bars = 20 μm. Data shown are representative of three independent experiments each performed with *n* = 4; images are consistent for a minimum of three micrographs per mouse.

Lung tissue from the same PBS and OVA treated *Il33^Cit/+^* reporter mice was cryosectioned. Sections were stained with DAPI (blue) to identify nuclei. WT mice showed no background citrine fluorescence and PBS-treated *Il33^Cit/+^* reporter mice showed very little fluorescence, though upon increasing the gain used in visualising the PBS-treated *Il33^Cit/+^* mouse lung sections the constitutive citrine fluorescence could be detected (Fig. 1D). By contrast, when the mice were treated with OVA, numerous bright citrine^+^ cells were visible (Fig. 1D). Strikingly, the cells producing citrine were not bronchial or bronchiolar epithelial cells, as suggested in previous reports 10,27,28. DAPI staining and smooth muscle actin staining were used to define airways and blood vessels (Fig. 1D). Further phenotypic analysis of the citrine^+^ epithelial cells by flow cytometry revealed expression of the type-2 pneumocyte marker, CD138 (syndecan-1) (Fig. 2A) 29. Isotype controls are shown in Supporting Information Fig. 1D. Citrine fluorescence co-localised with surfactant protein C (SPC), another marker of type-2 pneumocytes (Fig. 2B) 30. To validate the *Il33^Cit/+^* reporter and to assess the localisation of IL-33 protein within the cell, *Il33^Cit/+^* lung sections from OVA-treated mice were stained with an anti-IL-33 antibody. Confocal images demonstrated co-localisation of the *Il33^Cit/+^* reporter and IL-33 protein (Fig. 2C and Supporting Information Fig. 2). Notably, IL-33 protein localised to the nucleus (Fig. 2C).

A kinetic study of the OVA response was conducted to investigate the temporal expression profile of IL-33. *Il33^Cit/+^* reporter mice were sensitised with two i.p. injections of OVA/Alum followed by either one or three nebulisations of 1% OVA, to compare to the original six nebulisations. Twelve hours after the last nebulisation, mice were sacrificed and tissues taken for flow cytometric analysis and cryosectioning. A significant upregulation of citrine production was seen after one nebulisation of OVA (over 7-fold above the PBS control) and this increased over the duration of the experiment (Fig. 2D). The population of IL-33 expressing type-2 pneumocytes became more dominant with increasing OVA exposure (Fig. 2D). Confocal imaging of the OVA-treated lungs confirmed this increase in the number of type-2 pneumocytes expressing citrine (Fig. 2E). After six nebulisations the major citrine producers were type-2 pneumocytes. The intensity of citrine expression increased over the time course, as did that of SPC expression (Fig. 2E).

### Induction of IL-33 promoter-driven citrine expression in the lung by pollen allergens

**Figure 3:**
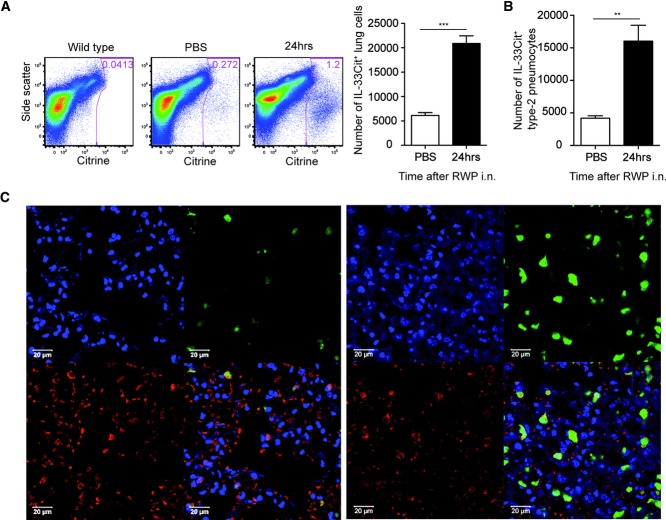
Ragweed upregulates IL-33 promoter expression. (A) Tissue from lungs of *Il33^Cit/+^* mice treated with either ragweed or PBS were stained for flow cytometry to assess citrine expression. (B) Expression of citrine by lung cells and by type-2 pneumocytes was assessed by flow cytometry. Data are shown as mean + SEM of *n* = 3–4 and are representative of three independent experiments. **p < 0.01; ***p < 0.001, unpaired Student's *t*-test. (C) A confocal image of a cryosection of lung tissue from an *Il33^Cit/+^* (green) mouse treated with either PBS (left) or ragweed (right) and stained with DAPI (blue) and SPC (red) is shown at 63× original magnification. Scale bar = 20 μm. Data shown representative of two independent experiments each performed with *n* = 3; images are consistent for minimum of three micrographs per mouse.

Ragweed pollen is a major cause of allergic asthma in North America. However, little is known about the mechanism for its involvement. To investigate this pathway in vivo a short-ragweed protein (RWP) challenge model was used to induce allergic lung inflammation in *Il33^Cit/+^* mice. Four intranasal doses of RWP were given once daily and 24 h after the fourth day of administration, citrine expression was upregulated (Fig. 3A). After ragweed treatment 80 000 citrine^+^ cells were observed in lung tissue. A significant proportion of citrine producers identified were type-2 pneumocytes (CD45^−^, EpCam^+^, CD138^+^) (Fig. 3B). The remaining RWP-induced citrine^+^, cells, as in the response to OVA, included alveolar macrophages, DCs, eosinophils and B cells. After treatment with ragweed the number of type-2 pneumocytes producing citrine, increased approximately 4-fold (Fig. 3B). Confocal imaging of lung sections show that RWP treatment increases the frequency and intensity of citrine producing cells. Confocal imaging confirmed that a proportion of citrine^+^ cells were SPC-expressing type-2 pneumocytes surrounding the alveoli (Fig. 3C).

### Induction of IL-33 promoter-driven citrine expression in the lung by fungal allergens

**Figure 4:**
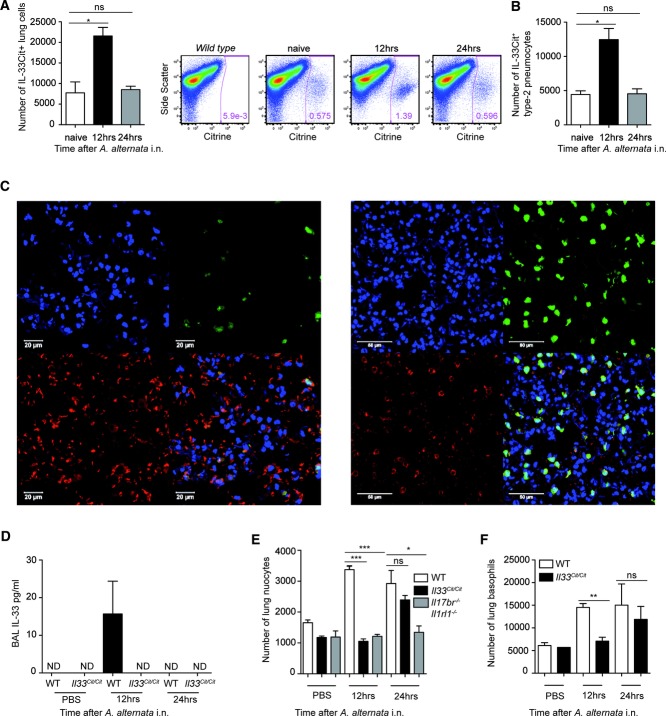
*Alternaria alternata* upregulates IL-33 promoter expression. (A) Expression of citrine after intranasal *A. alternata* administration was determined by flow cytometry. (B) Citrine expression by type-2 pneumocytes (CD45^−^/EpCam^+^/CD138^+^) was determined by flow cytometry and shown as mean + SEM of *n* = 3–5 mice and representative of three experiments performed. (C) Confocal images of cryosections of *Il33^Cit/+^* (green) lung tissue treated with PBS (left) or *A. alternata* (right) and stained with DAPI (blue) and SPC (red) are shown. Data shown are representative of three independent experiments each performed with *n* = 3–5 mice. (D) IL-33 protein levels in the BAL of WT or *Il33^Cit/Cit^* (*Il33^−/−^*) mice 12 or 24 h after *A. alternata* administration were measured by ELISA (ND: not detected). (E, F) The frequency of (E) nuocytes or (F) basophils in WT or IL-33-deficient mice (*Il33^Cit/Cit^*) or IL-25 receptor and IL-33 receptor deficient mice (*Il17br^−/−^Il1rl1^−/−^*), 12 or 24 h after *A. alternata* or PBS administration was determined by flow cytometry. Data are shown as mean +SEM of *n* = 3–4 and are representative of two independent experiments. *p < 0.1; **p < 0.01; ***p < 0.001, unpaired Student's *t*-test. ns: not significant.

It is known that airborne allergens induce allergic lung inflammation and recently this has been linked to IL-33 induction 31. *Il33^Cit/+^* mice were treated with culture filtrate extracts of the fungal pathogen *Alternaria alternata* 31. Twelve hours after a single *A. alternata* dose a marked upregulation of citrine expression was observed (approximately 3- to 6-fold), which also coincided with an increase in the MFI of the citrine-producing cells. However, 24 hours after dosing the level of citrine expression had almost returned to that of the naïve mice (Fig. 4A). At both time points after challenge, CD45^−^ cells were the major citrine producers. In naïve mice, almost no CD45^+^ cells expressed citrine; however, after treatment this population increased in size and included alveolar macrophages, eosinophils, DCs and B cells (data not shown). The majority of the citrine^+^ CD45^−^ cells identified expressed EpCam and CD138, defining them as type-2 pneumocytes; their numbers peaked 12 h after dosing (Fig. 4B). Confocal images of lung sections stained for SPC confirmed the presence of IL-33 producing type-2 pneumocytes and illustrated the increased number and intensity of citrine-producing cells 12 h after dosing (Fig. 4C).

To assess the role of IL-33 production by pneumocytes upon *A. alternata* exposure an IL-33-deficient mouse (*Il33^Cit/Cit^*) was generated and treated with *A. alternata*. IL-33 protein was detected in the bronchoalveolar lavage (BAL) of WT mice at 12 h after dosing with *A. alternata*, but could not be detected in *Il33^Cit/Cit^* mice (Fig. 4D). Reduced nuocyte and basophil numbers were observed in the IL-33-deficient mice 12 h after immunisation. However, after 24 h nuocyte and basophil numbers were equivalent to those seen in the WT counterparts (Fig. 4E and F). To assess the potential additional role of IL-25 in nuocyte proliferation *Il17Br^−/−^Il1rl1^−/−^* mice were also treated with *A. alternata*, at both 12 and 24 h. After *A. alternata* treatment nuocyte numbers were further reduced in the *Il17Br^−/−^Il1rl1^−/−^* mice compared to WT and *Il33^Cit/Cit^* mice, and indeed did not rise above PBS-challenged controls (Fig. 4E).

### Induction of IL-33 promoter-driven citrine expression

**Figure 5:**
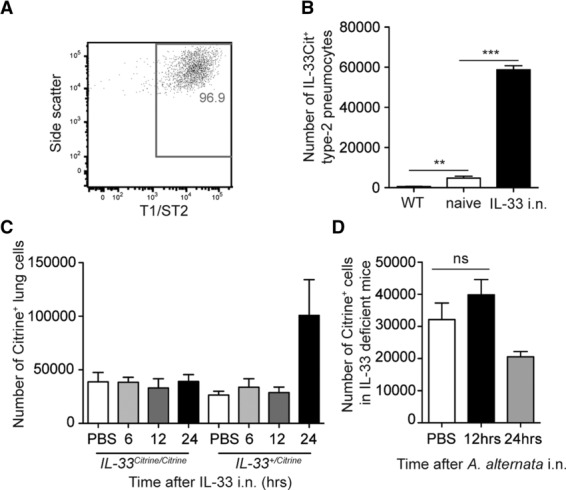
IL-33 upregulates its own production. (A) Type-2 pneumocyte expression of T1/ST2 was determined by flow cytometry. Data are representative of six independent experiments each performed with *n* = 5. (B) The frequency of citrine^+^ type-2 pneumocytes in the lung tissue of mice treated with IL-33 was determined by flow cytometry. Data are shown as mean +SEM of *n* = 4 and are representative of two independent experiments. (C) The frequency of citrine^+^ lung cells in either *Il33^Cit/Cit^* or *Il33^Cit/+^* mice after intranasal IL-33 administration is shown. (D) The frequency of citrine^+^ lung cells in *Il33^Cit/Cit^* mice after *A. alternata* administration is shown. (C, D) Data are shown as mean +SEM of *n* = 3–4 and are representative of two independent experiments.

Notably, we identified that T1/ST2 (isotype control within Supporting Information Fig. 1D) is expressed on citrine-expressing pneumocytes (Fig. 5A) and that citrine expression clearly marks those cells positive for IL-33 protein expression (Fig. 2C). This raised the question as to whether IL-33 might act in an auto/paracrine manner. The effect of IL-33 on type-2 pneumocytes was assessed by administering IL-33 intra-nasally to *Il33^Cit/+^* reporter mice and subsequently analysing lung tissue expression of citrine. Three doses of IL-33 alone induced an approximately 12-fold increase in citrine production by type-2 pneumocytes (Fig. 5B). Thus, IL-33 (or a signal downstream of IL-33) has the capacity to promote IL-33 expression, potentially via an auto/paracrine response through its receptor expressed on pneumocytes. To test this we administered a single dose of IL-33 intra-nasally to *Il33^Cit/Cit^* mice, which are IL-33-deficient, and *Il33^Cit/+^* mice to determine whether the ability of the mice to express endogenous IL-33. Notably, at this lower dose of IL-33 only mice able to produce endogenous IL-33 induced a detectable increase in citrine expression (Fig. 5C and Supporting Information Fig. 3A). To further investigate this possibility, *Il33^Cit/Cit^* mice, were treated with *A. alternata* and the production of citrine by lung epithelial cells was assessed 12 and 24 h later. We hypothesised that if IL-33 were required for its own upregulation, then in the absence of IL-33, fluorescent reporter expression would not be amplified in response to *A. alternata*, in contrast to the response in IL-33 sufficient mice (Fig. 4B). We found that in the *Il33^Cit/Cit^* mice there was an impairment in the upregulation of citrine 12 h after *A. alternata* administration (Fig. 5D and Supporting Information Fig. 3B), compared to the response in *Il33^Cit/+^* mice, which have one functional IL-33 allele (Figs. 4B and 5D). This suggests that in the absence of IL-33 there may be a failure to feedback and amplify the IL-33 response.

## Discussion

IL-33 can act as a histone-binding factor and a cytokine. By binding to its cognate receptor, T1/ST2, IL-33 initiates immune pathways such as the type-2 immune response in allergic asthma. Neither of these functions of IL-33 has been fully explained nor have the cellular sources of the cytokine been fully determined. This report defines in vivo cellular sources of IL-33 in both naïve mice and mice undergoing allergic lung responses. Constitutive expression of IL-33 was seen in the lungs of naïve mice, with 0.1% of lung cells producing citrine. This expression could represent the transcriptional requirement for maintenance of the stored IL-33 levels, available to be released as an alarmin upon cell damage. IL-33 production was also observed in naïve draining lymph nodes.

The OVA model is widely used to induce experimental allergic lung inflammation to study immunopathology in mice. This report shows that IL-33 expression is strongly upregulated during the OVA response. Upon OVA treatment both the fluorescence intensity and number of epithelial cells in the lung tissues expressing IL-33 were highly upregulated. Indeed, 5% of all lung cells produced citrine upon OVA stimulation. We also observed an increase in citrine expression when mice were only treated with PBS. This could be due to the mechanical effect of the liquid/vapour instillation into the lung. Indeed, Kakkar et al. 32 reported that IL-33 expression is induced by mechanical stresses, as well as allergens or other immune stimuli.

Visualisation of the inflammatory response using confocal microscopy clearly indicated that not all types of airways epithelial cells upregulated IL-33 expression. Notably, the cells lining the bronchioles were not citrine-positive. Our findings in vivo support the conclusions made by Kim et al. 16 in epithelial cell lines, where they detected no IL-33 mRNA from MLE12 airways epithelial cells. Also in support of the in vitro conclusions of Kim et al. 16, our staining of cryosections, and flow cytometric analysis, using anti-SPC and anti-CD138 antibodies, show that when induced in vivo by either OVA, ragweed or fungal extract, it is type-2 pneumocytes specifically that produce IL-33. These results, combined with those of Yasuda et al. 33 showing IL-33 production from type-2 pneumocytes after nematode infection, confirm alveolar epithelial cells as important immune cells in lung immune responses. Type-2 pneumocytes make up 60% of the epithelial cells that line alveolar spaces in the lung tissue. They secrete surfactant, which reduces alveolar surface tension and maintains ease of breathing by preventing collapse of the air spaces 16. To fulfil their role, as the gas exchange units of the lung, the alveoli create a vast surface area. The alveolar walls alone account for over 99% of the lung's internal surface area 32 and as such the type-2 pneumocytes are perfectly positioned for sensing allergens and secreting IL-33 to instigate the type-2 response in allergic asthma. Analysis of the type-2 pneumocytes showed that in addition to producing IL-33 they also expressed T1/ST2, thus making them capable of responding to IL-33 produced by type-2 pneumocytes themselves or by other cells such as the CD45^+^ IL-33-producing cells observed in the lung. This potentially implicates an auto/paracrine propagation of IL-33 signals, which may explain the significant increase in citrine expression later in the OVA response. Not only did the level of expression of IL-33 (fluorescence intensity) increase over the time course, but that of SPC also increased. This potentially implies that alveolar epithelial cells produce more surfactant during allergic lung inflammation, possibly in an attempt to restore ease of breathing or bind the allergen and prevent further reaction. A mutation in SPC has been linked to an increased risk of asthma 33.

This report along with those of other groups 16,34 clearly shows IL-33 expression from the alveolar epithelium, but not from the epithelial cells of larger airways. This is in contrast to that observed in the human lung where IL-33 has been reported to be expressed in larger airway bronchial epithelial cells 28. It is therefore important to consider the species-specific differences in IL-33 expression if targeting such production therapeutically.

Aeroallergens trigger type-2 immune responses in the lungs of asthma sufferers. However, the mechanisms behind the recognition of allergen and subsequent initiation and maintenance of the immune response remain to be fully elucidated. In this study we used two different aeroallergens, *A. alternata* and ragweed pollen to induce a type-2 response in the lung and studied the cytokines involved. Extract from *A. alternata* has been shown to induce an increase in the level of IL-33 in the lungs of treated mice 31 and innate lymphoid cells have been shown to be responsive to this IL-33 expression 35. Here we show for the first time in vivo that EpCam^+^ CD138^+^ type-2 alveolar epithelial cells produce IL-33 in response to *A. alternata* and ragweed pollen. The response to ragweed elicits expression of IL-33 promoter-driven citrine from a larger CD45^+^ cell population than observed in the OVA response. This may simply be due to the time of analysis, since the OVA model requires pre-sensitisation with antigen, whilst *Alternaria* does not. The response to *A. alternata* is short lived, the expression level of citrine peaks at 12 h and returns to the naïve level after 24 h. This result supports the findings of Wills-Karp et al. 24 that IL-33 is transiently expressed with protein levels peaking one day after *N. brasiliensis* infection and places IL-33 at the initiation of type-2 responses. Use of the IL-33-deficient mouse showed that without IL-33, the innate type-2 response to *A. alternata*, including expansion of the nuocyte population, was delayed. Nuocytes, producing IL-13, have been shown to be essential for the development of airways hyper-reactivity during experimental allergic lung inflammation 14 and here we show IL-33 is required for a rapid expansion of this critical cell population. Twelve hours after immunisation WT mice demonstrate significant expansion of the lung nuocyte and basophil populations, correlating with detection of IL-33 in the BAL, whereas these cells failed to expand in the IL-33-deficient mice. However, by 24 h after immunisation, the numbers of nuocytes and basophils were comparable in WT and IL-33-deficient mice. These data indicate that IL-33 has an early critical role in rapidly responding to allergens and eliciting nuocyte expansion. However, in its absence there is redundancy in the type-2 response. Nuocyte numbers, though delayed, eventually rise in IL-33-deficient mice to those observed in WT animals, this was not the case in mice that could not respond to either IL-33 or IL-25.

Administration of three doses of IL-33 alone induced a significant increase in citrine production by type-2 pneumocytes. In the absence of any allergenic component, that could, e.g. stimulate PAMP receptors, exogenous IL-33 induced upregulation of IL-33 by type-2 pneumocytes. Thus damage, by, e.g. the protease action of certain allergens, may release IL-33 from cells that acts via T1/ST2 on type-2 pneumocytes (or other cells), inducing upregulation of IL-33 and possibly feeding into a propagation loop. We noted that when we administered a lower dose of exogenous IL-33 only mice capable of producing endogenous IL-33 went on to activate the IL-33 promoter, whilst IL-33-deficient (*Il33^Cit/Cit^*) mice did not, implying that the additional endogenous IL-33 was necessary to potentiate the IL-33 pathway. This release and feedback would lead to amplification of the response and may explain how IL-33 acts as an alarmin. Although we also observed an increase in the number of citrine-positive cells detected in the lungs of *Il33^Cit/Cit^* mice, as compared with that in *Il33^Cit/+^* mice, this may be explained by the increase in citrine MFI when 2 *Citrine* alleles were present leading to cells from the *Il33^Cit/Cit^* mice being brighter than their heterozygous counterparts and being included in the positive gate for citrine expression. A further possibility is that cell death induced by *A. alternata* treatment may have resulted in reduced numbers of IL-33 positive cells (as compared with that resulting from PBS administration) and that without paracrine feedback new IL-33 positive cells were not elicited.

Our data, using *Il33^Cit/+^* reporter mice, identify sources of IL-33 in naïve mice. Using experimental models of allergic asthma we describe the temporal and spatial expression profile of IL-33 in vivo during type-2 lung inflammatory responses. We show that IL-33 is required for the rapid expansion of nuocytes, which express high levels of IL-5 and IL-13 and contribute to the asthmatic response 14. These findings give us new insights into the initiation and development of experimental lung inflammation and provide new cellular targets for improving our understanding of the allergic asthma pathway.

## Materials and methods

### Mice

The gene encoding citrine fluorescent protein was targeted to the *Il33* locus in murine BALB/c embryonic stem cells using recombineering. A 7 kb genome DNA fragment centred on the *Il33* start codon in exon 2, was amplified by PCR with primers: aseq4410 (agctctccaccgcggccgcctgcaggaaaagtcagcattc), introducing a Not1 site and aseq4411 (taagtgatcctagggct gtggccacccaatgg) and cloned into bluescript SK^−^ plasmid. The fluorescent citrine cassette was recombineered into the *Il33* gene just after the start codon of the gene using primers aseq4446 (ggtccatatagttggattattgttatatttcaatcccacaga aacctgaaaaatggtgagcaagggcgaggagctgttcaccgg) and aseq4448 (cgcccgtcttcatgttgaaataacaaatatttgataatagatttaatagtggata,acttcgta tagcatacattatacg) and in doing so 113 bp of the *Il33* gene immediately downstream of the start codon were removed (Supporting Information Fig. 1A). The screening probe was amplified by PCR from genomic DNA using primers aseq4406 (gatacgtgtgtgacatagcc) and aseq4407 (acacagtcgcgcttcagttc). Success of embryonic stem cell recombination and later success of germline transmission was determined by Southern blot. Chimeric mice that were obtained were crossed with BALB/c mice to obtain germline transmission. *Il33^Cit/+^* mice were genotyped by PCR using primers aseq4432 (caaggcagtgccgataaagg) and aseq863 (cttgggtggagaggctattc) the citrine^+^ product being 1625 bp and with aseq4708 (ccagatgaacttgtgattgttgctccctc) and aseq4709 (cttggagttggaatacttcatt ctagg) the WT product being 1000 bp. The reporter mice were crossed with BALB/c cre deletor mice (that had been back-crossed for more than ten generations), to remove the neomycin gene used for clonal selection during the generation process. *Il33^Cit/+^* mice were inter-crossed to obtain *Il33^Cit/Cit^* functional *Il33* knockout mice.

BALB/c (Charles River facility, 6–8 weeks old) and the above-mentioned transgenic mice were maintained in specific pathogen-free environments. All experiments were undertaken with the approval of the UK Home Office.

### Substance administration

Mice were lightly anaesthetised with isoflurane and given intra-nasally either: 25 μg *A. alternata* culture filtrate extract (Greer Laboratories), 100 μg of short RWP (Greer Laboratories), on days 0, 1, 2 and 3 or 0.5 μg of recombinant mouse IL-33 on days 0, 1 and 2 (or the equivalent dose of PBS). Mice were sacrificed and tissues taken 12 and 24 h after challenge.

### Sensitisation and OVA exposure

Mice were sensitised with OVA according to our previous reports 36. Mice were then sacrificed 12 h after the last lung challenge and tissues were taken for analysis.

### Flow cytometry

Lungs were treated with 720 μg/mL collagenase D (Amersham, Bucks, UK) and then lymph node and lung cell suspensions were incubated with anti-Fc receptor blocking antibody (anti-CD16/CD32 (93)). Cell-surface marker expression was assessed with mAbs against CD11c (N418), CD11b (M1/70), Siglec-F (E50–2440), Gr-1 (RB6–8C5), CD45 (30-F11), EpCam (G8.8), CD138 (281–2), T1/ST2 (DJ8), CD3 (145–2C11), CD4 (30F11), CD8 (53–6.7), CD19 (1D3), FcεR1 (MAR-1), CD49b (DX5) or ICOS (C398.4A). Isotype and single-stain controls were included. The samples were acquired by using an LSRII (Becton Dickinson, Franklin Lakes, NJ) and analysed with FlowJo software (version 9.2).

### Confocal microscopy

Lobes of lung tissue were incubated in 4% PFA solution and then in 20% sucrose before being frozen in OCT or gelatin. Frozen tissues were then sectioned and stained with DAPI, Cy3-conjugated anti-smooth muscle actin, pro-SPC (Millipore AB3786) and anti-mouse IL-33 (R&D systems) with an Alexa647 secondary antibody. Images were taken on a Carl-Zeiss inverted microscope (LSM 710) and processed with ZEN 2008 (Carl-Zeiss).

### Detection of IL-33 protein

IL-33 protein levels in the BAL were detected by ELISA (Quantikine Mouse IL-33 kit R&D).

### Statistical analysis

Student unpaired *t*-tests analysis were performed.

## Abbreviations

BAL: bronchoalveolar lavage
***IL-33^Cit/+^***: interleukin-33 citrine reporter mouse line
RWP: ragweed protein
SPC: surfactant protein C

## Supplementary material

As a service to our authors and readers, this journal provides supporting information supplied by the authors. Such materials are peer reviewed and may be re-organized for online delivery, but are not copy-edited or typeset. Technical support issues arising from supporting information (other than missing files) should be addressed to the authors.
