# Supplementary material for: IL-33 citrine reporter mice reveal the temporal and spatial expression of IL-33 during allergic lung inflammation
Source: Eur J Immunol. 2012 Nov 21;43(2):488–98. doi: 10.1002/eji.201242863 (PMC3734634; doi:10.1002/eji.201242863)
Supplement: Supplementary file 2 [file eji0043-0488-SD2.pdf]

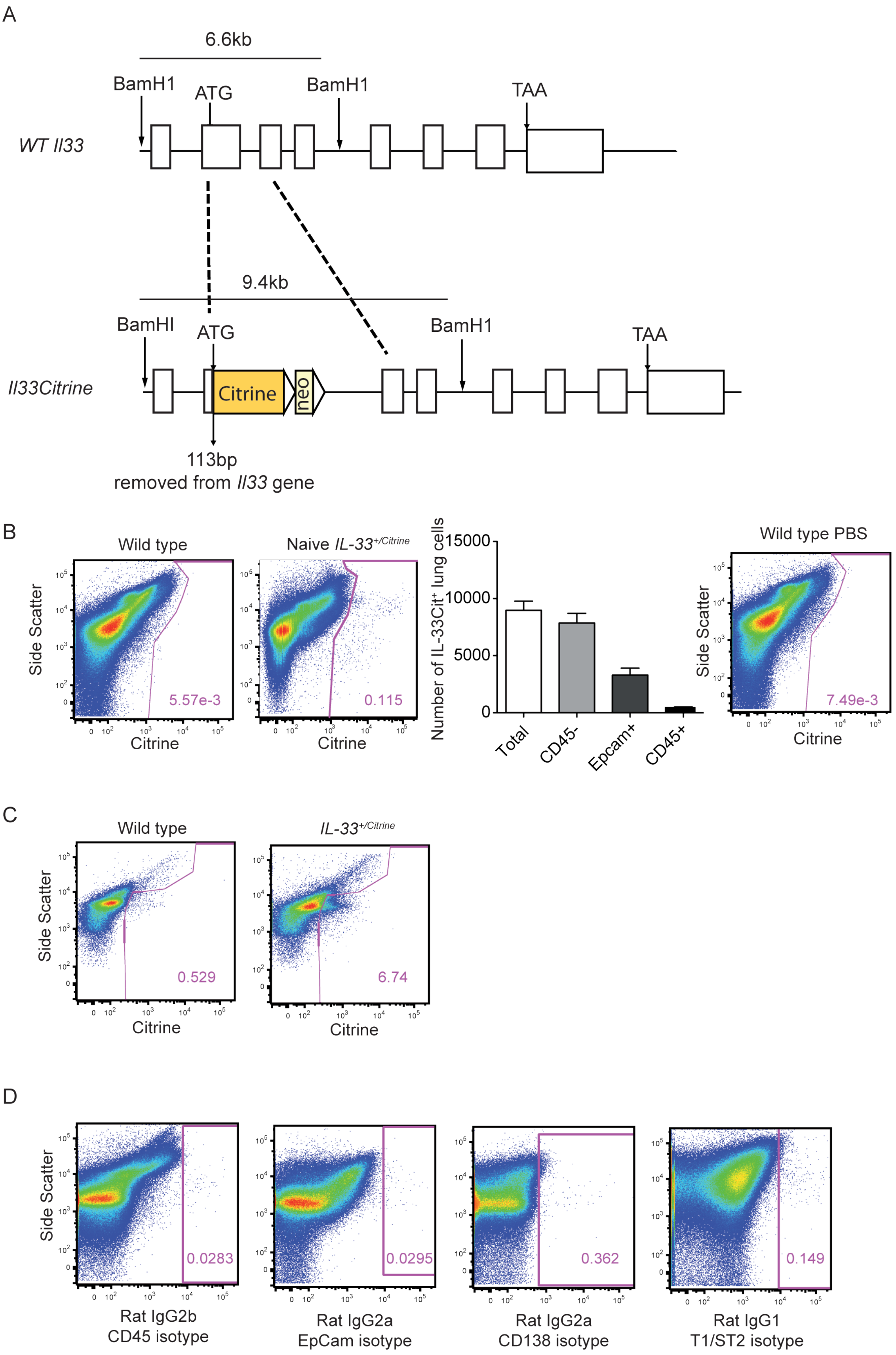

Supporting Information Figure 1. Generation of an IL-33Citrine reporter and defining naïve IL-33 expression. (A) Schematic of *Citrine* insertion into the *Il33* gene immediately down stream of the start codon. (B) Flow cytometry data of citrine expression in the lung tissue of naïve mice and citrine+ cells' expression of epithelial cell markers n = 3 - 4. Data are representative of 6 independent experiments. Error bars represent mean+/- SEM. (C) Flow cytometry data of citrine expression in the mediastinal lymph node tissue of naïve mice n = 4. Data are representative of 2 independent experiments. D. flow cytometry isotype controls for pneumocyte gating strategy i) CD45 ii) EpCam iii) CD138 iv) T1/ST2.

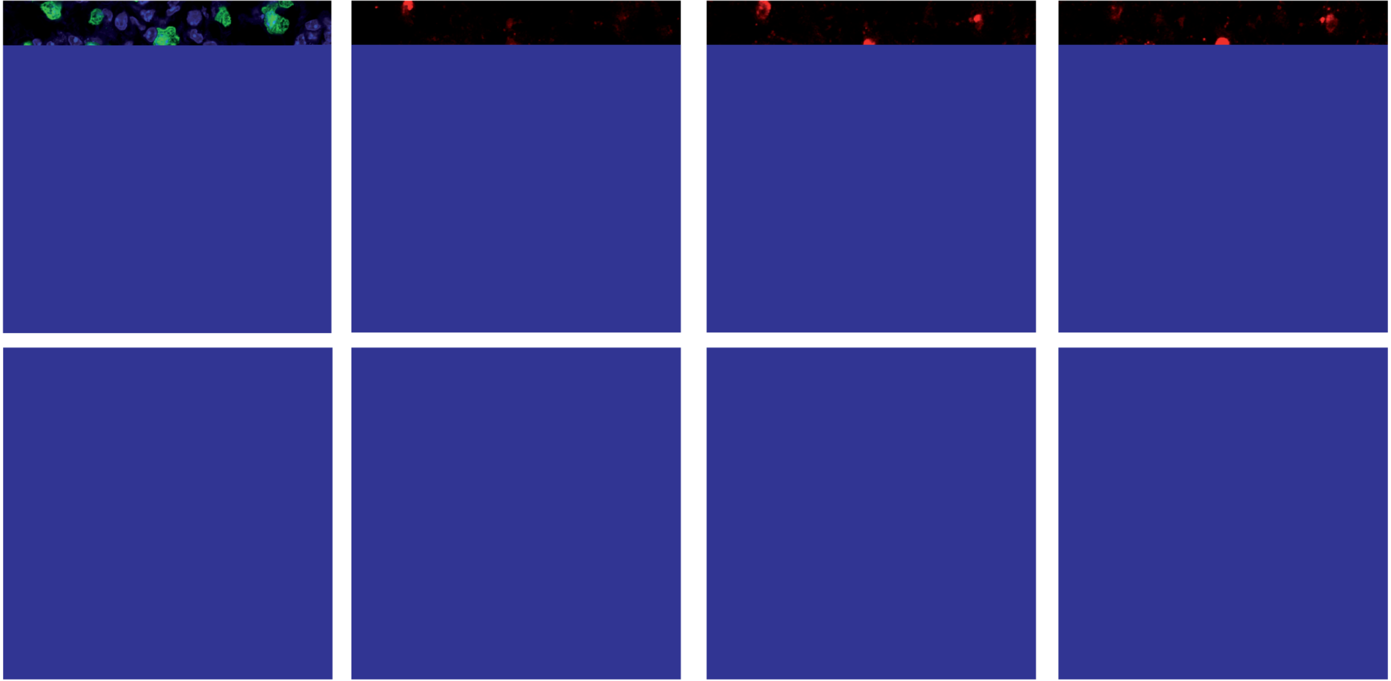

Supporting Information Figure 2. Colocalisation of citrine and IL-33 antibody fluorescence.
Confocal image (63x) of lung citrine expression following OVA treatment, citrine (green) and DAPI (blue). With a 7-panel Z-stack (63x) showing anti IL-33 antibody staining (red) for the same field of view.
